# Survey data on gender in relation to youth political discussion and involvement at a Public University in Ghana

**DOI:** 10.1016/j.dib.2020.105796

**Published:** 2020-06-03

**Authors:** Olusegun Sunday Ewemooje, Acheampong Yaw Amoateng, Elizabeth Biney

**Affiliations:** a Population and Health Research Entity, Faculty of Humanities, North-West University (Mafikeng Campus), North West, South Africa; b Department of Statistics, Federal University of Technology Akure, Akure, Nigeria

**Keywords:** Gender, Political participation, Political involvement, Politics, Ghana, Youth

## Abstract

This article presents extensive description of survey data on the political participation of 913 male and female undergraduate students at the University of Ghana. Multi-stage and other sampling procedures were employed to collect the data that took place between 2016 and 2017. Data were analysed using frequencies, percentages and cross-tabulations for each gender separately. The findings revealed that females reported discussing politics more frequently with others than their male counterparts, in addition, ethnicity significantly associated with political discussion and religion significantly associated with political involvement for females. However, males expressed interest and involvement in political activities at both national and student levels more frequently than their female counterparts. The findings support much of the observations in the political behavior literature. Despite the gender imbalance, respondents were partial to engagement in student politics than national politics. It is, therefore, advisable that political parties focus their recruitment efforts on university campuses.

Specifications table

**Table:** 

Subject	Social Sciences, Humanities.
Specific subject area	Population Studies, Political Science, Political Sociology, Youth Studies
Type of data	Tables
How data were acquired	Information was gathered by administering semi-structured questionnaires to sampled students. A copy of the questionnaire is attached as a supplementary file.
Data format	Raw Analysed
Parameters for data collection	Information collected included sociodemographic characteristics, academic performance, religiosity, political knowledge and participation, substance use, as well as opinions on sex and sexuality (see supplementary files).
Description of data collection	Data were obtained from 913 undergraduate students contacted with the use of multi-stage sampling (stratified and systematic sampling techniques).
Data source location	The University of Ghana, Greater Accra Region, Ghana.
Data accessibility	Data are included in this article

## Value of the data

• The data offer vital information on individual background characteristics, opinions on the importance of participation in civic activities, as well as how much young people say they discuss politics and with whom. This can be useful for anyone who has an academic interest in political discussion, political participation and/or deliberative democracy beyond electoral politics (i.e. elections, voting), particularly among the youth population.
• The proclivity of citizens to engage in political discussion is considered to be a basic component of democratic political systems. Thus, the data can be useful in advancing the academic debate regarding the contribution of everyday political discussion in a democracy.
• This survey is primarily focused on two principal demographic variables, gender and age, for their consistent identification as important predictors in the political participation literature. This information can be used to examine the role of gender or age on political participation to corroborate or contradict the existing assertion that young people and women are particularly politically apathetic or apolitical [1].
• The data can be used to explore the relationship between socio-demographic variables and young people's political involvement, to draw inferences on how political participation is shaped by individual and contextual factors.
• The dataset is limited to Ghana, a fairly politically stable multi-party democracy with a young age structure. Thus, information derived from the data can be used to draw inferences about the link between young people's civic participation and democratic governance. It can also be used to provide anecdotal narratives for countries in transition or developing democracies in sub-Saharan Africa.
• The dataset also includes information on other variables that can guide further investigations into and/or comparative research into young people's perceptions of and attitudes toward premarital sex, substance use, and other prevalent issues in sub-Saharan Africa (see supplementary file) [2].

## 1 Data

Table 1 shows the background characteristics of the respondents. The age distribution of respondents shows that the lowest age is 17 years, the highest age is 45 years, with an average age of 21 years. Also, 267 (30.2%) are less than 20 years, 573 (62.8%) are between 20–24 years old, while 64 (7%) are 25 years or older. The gender disaggregation of respondents shows that 440 (48.2%) are male while 473 (51.8%) are female. More than half (58.7%) of the respondents identify as Akan, 12.1% are Ga-Adangbe, 15.6% are Ewe, while 13.6% belong to other ethnic groups. The majority (897;98.2%) of the respondents are Ghanaians, while only about 1.8% are foreign nationals. Just over two-sevenths (29.2%) of the respondents are in their second year, 17.1% are first-year students, 27.1% are third-year students and 26.6% are fourth-year students. Breakdown by respondents’ academic faculty shows that more than half (53.4%) are enrolled in the Humanities/Social Sciences, about a quarter (25.4%) are in the Basic and Applied Sciences, and about a fifth (20.9%) are enrolled in the Health Science faculty. Lastly, nearly half (48.3%) of the respondents reported coming from middle-income households, while 45% reported coming from richer households, with 6.7% coming from poorer households.

**Table 1:** Distribution of the respondents by sociodemographic characteristics.

	Frequency	Percent
Age Group		
<20 years	276	30.2
20–24 years	573	62.8
≥25 years	64	7.0
Gender		
Male	440	48.2
Female	473	51.8
ᶲEthnicity		
Akan	530	58.7
Ga-Adangbe	109	12.1
Ewe	141	15.6
Others	123	13.6
Nationality		
Ghanaian	897	98.3
Foreign nationals	16	1.7
Academic Year of Study		
First-year	156	17.1
Second-year	267	29.2
Third-year	247	27.1
Fourth-year	243	26.6
ᶲFaculty of Study		
Humanities/Social Sciences	486	53.6
Basic and Applied Sciences	231	25.5
Health Science	190	20.9
ᶲFamily SES		
Richer	404	45.0
Middle	434	48.3
Poorer	60	6.7
Total	913	100.0

ᶲ Variable has missing values.

Tables 2 and 3 present information on respondents’ political interest as measured by their tendency to discuss politics with others. Generally, the analysis shows a slight gender difference in the reported levels of political interest, in favor of females. Thus, females more frequently reported discussing politics with others compared to males. This observation contradicts prevailing assumptions that, compared with their female counterparts, males are more likely to engage in political discussions [3,4]. Additionally, ethnicity is significantly associated with political discussion for females. This suggests that for females, in particular, the ethnic group they belong to has important bearings on whether or not they engage in political discussions with other people. Perhaps there may be cultural norms or taboos relevant to certain ethnic groups that regulate the involvement of women in deliberative interactions. For instance, Ghanaian society is organised around either patrilineal or matrilineal lines. Therefore, in matriarchal ethnic societies (such as the Akans) women are considered equal to men and as such may be encouraged to express themselves in all matters as their male counterparts do.

**Table 2:** Relationship between students’ political discussion and socio-demographic factors by gender.

	Male				Female			
	No	Yes	Total	p-value	No	Yes	Total	p-value
Age				0.294				0.722
< 20 years	32.3%	67.7%	96		25.0%	75.0%	180	
20–24 years	26.7%	73.3%	315		26.7%	73.3%	258	
≥25	37.9%	62.1%	29		31.4%	68.6%	35	
Religion				0.789				0.240
Christian	29.3%	70.7%	375		27.1%	72.9%	398	
Muslim	20.0%	80.0%	20		20.8%	79.2%	24	
Others	23.5%	76.5%	17		66.7%	33.3%	3	
ᶲEthnicity				0.302				0.045
Akan	25.1%	74.9%	255		21.8%	78.2%	275	
Ga-Adangbe	30.4%	69.6%	56		26.4%	73.6%	53	
Ewe	36.1%	63.9%	72		37.7%	62.3%	69	
Others	30.0%	70.0%	50		30.1%	69.9%	73	
Academic Year of Study				0.223				0.196
First-year	25.8%	74.2%	62		25.5%	74.5%	94	
Second-year	34.5%	65.5%	116		26.5%	73.5%	151	
Third-year	30.5%	69.5%	128		32.8%	67.2%	119	
Fourth-year	23.1%	76.9%	134		20.2%	79.8%	109	
ᶲFaculty of Study				0.090				0.510
Humanities/Social Sciences	25.5%	74.5%	239		25.9%	74.1%	247	
Basic and Applied Sciences	37.6%	62.4%	117		31.6%	68.4%	114	
Health Science	25.6%	74.4%	82		22.2%	77.8%	108	
ᶲFamily Socioeconomic Status				0.668				0.355
Poorer	34.2%	65.8%	38		31.8%	68.2%	22	
Middle	27.3%	72.7%	220		29.0%	71.0%	214	
Richer	29.2%	70.8%	178		23.5%	76.5%	226	
Total	28.6%	71.4%	440		26.4%	73.6%	473	

ᶲ Variable has missing values.

**Table 3:** Gender disaggregation of proclivity to discuss politics with other people.

	Male						Female					
	Never	Rarely	Sometimes	Often	Always	Total Responses	Never	Rarely	Sometimes	Often	Always	Total Responses
Your father	25.6%	26.3%	28.3%	12.4%	7.3%	410	26.0%	28.9%	31.5%	9.2%	4.5%	447
Your mother	28.1%	30.9%	26.4%	9.4%	5.2%	424	18.2%	29.4%	36.4%	11.2%	4.8%	456
Family members other than parents	28.2%	26.8%	27.0%	13.5%	4.4%	429	30.2%	29.6%	24.8%	12.5%	2.8%	463
Friends/ classmates with different views	11.7%	15.7%	35.5%	22.7%	14.5%	428	10.8%	26.6%	36.0%	17.3%	9.3%	462
Friends and classmates with similar views	13.3%	17.3%	33.3%	21.3%	14.8%	427	12.6%	22.2%	35.7%	18.0%	11.5%	460
Members of political organisations on campus	44.2%	25.7%	14.2%	9.1%	6.8%	428	52.2%	23.3%	14.0%	6.5%	4.1%	464
Members of political organisations off campus	47.8%	24.8%	12.5%	8.6%	6.3%	431	55.5%	24.8%	12.3%	5.2%	2.2%	463

The rate of respondents’ active participation in various political activities by gender is presented in Tables 4 and 5. It is observed that generally, males are relatively more politically active than females, especially as it relates to influencing others’ political opinion, signing petitions, and raising political matters through letters. This supports similar observations in previous research [3,5]. However, the faculty and year of study are significantly associated with political involvement for females. It is probable that, because politics is generally seen as a male domain [3], the introduction of females to active politics is primarily and formally learned. Thus, there is a strong likelihood that females will become increasingly interested and involved in civic matters the more and longer they are exposed to it academically.

**Table 4:** Relationship between students’ active political involvement and socio-demographic factors by gender.

	Male				Female			
	No	Yes	Total	p-value	No	Yes	Total	p-value
Age				0.121				0.814
< 20 years	56.3%	43.8%	96		66.7%	33.3%	180	
20–24 years	50.8%	49.2%	315		64.0%	36.0%	258	
≥25	34.5%	65.5%	29		62.9%	37.1%	35	
Religion				0.381				0.233
Christian	50.4%	49.6%	375		65.6%	34.4%	398	
Muslim	45.0%	55.0%	20		54.2%	45.8%	24	
Others	70.6%	29.4%	17		100.0%	0.0%	3	
ᶲEthnicity				0.474				0.280
Akan	47.8%	52.2%	255		62.2%	37.8%	275	
Ga-Adangbe	58.9%	41.1%	56		66.0%	34.0%	53	
Ewe	52.8%	47.2%	72		75.4%	24.6%	69	
Others	52.0%	48.0%	50		63.0%	37.0%	73	
Academic Year of Study				0.499				0.047
First-year	45.2%	54.8%	62		55.3%	44.7%	94	
Second-year	56.0%	44.0%	116		68.9%	31.1%	151	
Third-year	48.4%	51.6%	128		71.4%	28.6%	119	
Fourth-year	51.5%	48.5%	134		60.6%	39.4%	109	
ᶲFaculty of Study				0.107				0.016
Humanities/Social Sciences	46.0%	54.0%	239		58.7%	41.3%	247	
Basic and Applied Sciences	57.3%	42.7%	117		74.6%	25.4%	114	
Health Science	56.1%	43.9%	82		69.4%	30.6%	108	
ᶲFamily Socioeconomic Status				0.845				0.932
Poorer	47.4%	52.6%	38		68.2%	31.8%	22	
Middle	50.5%	49.5%	220		64.5%	35.5%	214	
Richer	52.2%	47.8%	178		64.2%	35.8%	226	
Total	50.9%	49.1%	440		64.9%	35.1%	473	

ᶲ Variable has missing values.

**Table 5:** The level of active involvement in political activities by gender.

	Male						Female					
	Never	Rarely	Sometimes	Often	Always	Total Responses	Never	Rarely	Sometimes	Often	Always	Total Responses
Attempting to influence the political views of others	37.6%	22.8%	26.7%	9.3%	3.6%	439	42.7%	28.4%	22.6%	4.1%	2.1%	468
Writing letters to the newspapers about political matters	85.1%	9.4%	3.9%	1.1%	0.5%	437	89.5%	7.7%	2.6%	0.2%	0.0%	468
Presenting your views to politicians (e.g. by signing petitions)	76.5%	13.0%	6.8%	3.4%	0.2%	438	84.1%	9.0%	5.2%	1.5%	0.2%	466
Participate in the activities of a political party	64.1%	17.3%	13.1%	4.6%	0.9%	434	77.8%	11.9%	8.5%	1.1%	0.6%	469
Participate in political protest marches/political sit-ins/demonstrations	81.5%	10.5%	5.9%	1.6%	0.5%	437	90.2%	5.1%	3.2%	0.9%	0.6%	468
Attending political rallies	69.2%	13.2%	12.1%	4.1%	1.4%	439	81.2%	12.2%	5.6%	0.9%	0.2%	468
Participate in the activities in the youth movement of a political party	70.8%	11.9%	11.0%	5.9%	0.5%	438	79.5%	12.4%	6.2%	1.5%	0.4%	469
Attending any mass meetings/rallies dealing with student politics on campus?	65.8%	11.8%	14.1%	5.7%	2.5%	439	78.0%	12.6%	6.4%	2.6%	0.4%	468

Even so, both genders seem to be more active in student politics – i.e. participate in the activities of the youth movement of a political party as well as attend mass meetings or rallies dealing with student politics on campus – suggesting an unclear gender impact on political participation [6]. This suggests the need to target university campuses as they are seemingly fertile grounds for attracting more young people into the national formal political arena.

Tables 6 and 7 show gender differences in opinions on the importance of youth electoral participation. Most male and female respondents are of the view that youth involvement in electoral politics – national and local government elections and membership in student political associations – at both the national and student levels is very important. Thus, respondents possess a strong awareness of the importance of youth participation in democratic governance, a good criterion for developing political interest [7]. In particular, religion is significantly associated with political involvement for females. The role of religion in shaping female political participation but not male participation saliently alludes to the suppression of women in many religions. That is to say, religion also perpetuates the gender-based oppression in the larger society so religious attitudes and practices can delineate women's involvement in political matters.

**Table 6:** Relationship between the importance of youth political involvement and sociodemographic factors by gender.

	Male				Female			
	Not Important	Important	Total	p-value	Not Important	Important	Total	p-value
Age				0.520				0.467
< 20 years	14.6%	85.4%	96		15.0%	85.0%	180	
20–24 years	11.4%	88.6%	315		11.6%	88.4%	258	
≥25	17.2%	82.8%	29		17.1%	82.9%	35	
Religion				0.551				0.003
Christian	12.3%	87.7%	375		13.1%	86.9%	398	
Muslim	20.0%	80.0%	20		0.0%	100.0%	24	
Others	11.1%	88.9%	17		66.7%	33.3%	3	
ᶲEthnicity				0.110				0.751
Akan	10.2%	89.8%	255		12.4%	87.6%	275	
Ga-Adangbe	21.4%	78.6%	56		13.2%	86.8%	53	
Ewe	9.7%	90.3%	72		17.4%	82.6%	69	
Others	14.0%	86.0%	50		13.7%	86.3%	73	
Academic Year of Study				0.394				0.959
First-year	14.5%	85.5%	62		12.8%	87.2%	94	
Second-year	15.5%	84.5%	116		14.6%	85.4%	151	
Third-year	8.6%	91.4%	128		12.6%	87.4%	119	
Fourth-year	12.7%	87.3%	134		12.8%	87.2%	109	
ᶲFaculty of Study								
Humanities/Social Sciences	11.7%	88.3%	239		10.9%	89.1%	247	
Basic and Applied Sciences	16.2%	83.8%	117		14.0%	86.0%	114	
Health Science	9.8%	90.2%	82		16.7%	83.3%	108	
ᶲFamily Socioeconomic Status				0.081				0.444
Poorer	23.7%	76.3%	38		4.5%	95.5%	22	
Middle	11.8%	88.2%	220		13.1%	86.9%	214	
Richer	10.7%	89.3%	178		14.2%	85.8%	226	
Total	12.5%	87.5%	440		13.3%	86.7%	473	

ᶲ Variable has missing values.

**Table 7:** Respondents’ opinion on the importance of youth involvement in electoral politics.

	Male						Female					
	Not at all important	Slightly Important	Neutral	Moderately Important	Very Important	Total Responses	Not at all important	Slightly Important	Neutral	Moderately Important	Very Important	Total Responses
Elections for national government	3.7%	4.8%	13.7%	16.2%	61.6%	437	4.0%	4.9%	18.7%	14.6%	57.7%	471
Elections for local government	3.7%	5.5%	15.2%	23.7%	52.0%	435	4.5%	8.3%	22.2%	19.8%	45.2%	469
Student political associations	5.1%	7.6%	25.0%	26.9%	35.4%	432	4.5%	8.9%	21.7%	27.0%	37.9%	470

Active participation by all members of a society in the political process is an essential part of a functioning democracy [3,8]. A cursory analysis of the data reveals slight gender variations in the political involvement of young people in Ghana that can have implications on political equality. Since the sampled data is limited to one public university, it may not be advisable to generalize findings to all young people in Ghana or even all universities in the country.

## 2 Experimental design, materials, and methods

The data forms part of the Religion and Positive Youth Development Project, an initiative of the North-West University (Mafikeng Campus) and the University of Ghana. This specific dataset is solely focused on undergraduate students from the University of Ghana. Multi-stage cluster sampling procedures were employed in selecting the sample population. At the first stage, a probability sampling method, stratified sampling, was used to divide the sample by faculties – Humanities/Social Sciences, Basic and Applied Sciences and Health Sciences were selected – using proportional allocation to size based on the population of students in each faculty. Departments were selected from each faculty; from each department, core courses at all levels were selected for inclusion. Thus, the student population of each faculty determined the proportion of students to select for inclusion in the final sample. Each faculty samples were disaggregated by year of study. The sample numbers allocated to each year of study were random whereby trained field assistants surveyed the number of students in each faculty, department, and gender as determined a priori through the stratified random sampling procedure.

A total of 920 semi-structured questionnaires were administered to students in English, with a response rate of 99.2 percent (913). Data collection occurred between September 2016 and March 2017. Respondents were subjected to a series of questions relating to issues such as religiosity, sexuality, political participation, and attitudes towards and use of various licit and illicit substances. Since the questionnaires were anonymous, some respondents may have been reluctant to respond to some of the items, which led to missing values in some of the variables as indicated in the tables presented.

The data were descriptively and inferentially analysed into tables using frequencies, percentages and cross-tabulations with the use of Pearson Chi-Square test to determine the relationship between political involvement and socio-demographic factors. It is important to note that, for ease and clarity, composite variables were created for the analyses in Tables 2,4 and 6. This was done by collating the responses of the relevant indicators into a single variable. Thus, in Tables 2 and 4, the responses “never” and “rarely” were collated as one group “No” while the “sometimes”, “often” and “always” responses were collated as another group “Yes” for all the indicators to generate the binary responses for the composite variables. A similar approach was used to arrive at the binary responses in Table 6.

## Declaration of Competing Interest

The authors declare that they have no known competing financial interests or personal relationships that could have appeared to influence the work reported in this paper.

## Appendix Supplementary materials

Image, application 1

Image, application 2

Image, application 3
