# Supplementary material for: Survey data on gender in relation to youth political discussion and involvement at a Public University in Ghana
Source: Data Brief. 2020 Jun 3;31:105796. doi: 10.1016/j.dib.2020.105796 (PMC7300124; doi:10.1016/j.dib.2020.105796)
Supplement: Supplementary file 1 [file mmc1.docx]

**SPIRITUALITY, RELIGIOSITY AND POSITIVE YOUTH DEVELOPMENT AT THE UNIVERSITY OF GHANA, LEGON, GHANA**

**University of Ghana**

**JULY 2016**

| Respondent’s name | |  | | | | | | | | | | | | Interview time |
| --- | --- | --- | --- | --- | --- | --- | --- | --- | --- | --- | --- | --- | --- | --- |
| Respondent’s cell number | |  |  | |  |  |  |  |  | |  |  |  |  |
| Interviewer number |  |  | | Checked | |  | | Back checked | |  | | | | Date |

1. **How old are you? ***(Record age in complete years)*:**
2. **Are you male or female?**

| Male | 1 | Female | 2 |
| --- | --- | --- | --- |

1. **Which type of school did you attend for Senior Secondary School? ***Single response***

| Public school in urban area | 1 |
| --- | --- |
| Public school in rural area | 2 |
| Private school | 3 |
| Other (please specify) | 5 |

1. *****Ask participant’s ethnicity***

| Akan | 1 |
| --- | --- |
| Ga-Adangbe | 2 |
| Ewe | 3 |
| Guan | 4 |
| Mole-Dagbani | 5 |
| Other: (specify)................................... | 6 |

1. **What is your nationality? ***Single response***

| Ghanaian | 1 |
| --- | --- |
| Other West African countries | 2 |
| Rest of Africa | 3 |
| Other: Specify | 4 |

1. **What is your academic year of study? ***Single response***

| First year | 1 |
| --- | --- |
| Second year | 2 |
| Third year | 3 |
| Fourth year |  |

1. **What was your first year of registration for the above qualification?**

| 2 | 0 |  |  |
| --- | --- | --- | --- |

1. **In which College are you registered as a student? ***Single response***

| College of Humanities/Social Sciences | 1 |
| --- | --- |
| College of Basic and Applied Sciences(Agriculture/Veterinary Sciences/Biological/Physical Sciences) | 2 |
| College of Education/Extra Mural/Distance Education | 3 |
| College of Health Science(Medicine/Clinical Sciences | 4 |
| Other (specify) | 5 |

1. **Which of the following applies to you? In the semester the AVERAGE mark for ALL my subjects was... ***Single response***

| Below 40% | 1 |
| --- | --- |
| 40%-49% | 2 |
| 50%-59% | 3 |
| 60%-69% | 4 |
| 70%-74% | 5 |
| 75% or more | 6 |

**10. What language do you (MOSTLY) PREDOMINANTLY SPEAK at home? ONE ANSWER ONLY**

| English | 1 |
| --- | --- |
| Twi | 2 |
| Ga | 3 |
| Ewe | 4 |
| Mole/Dagbani | 5 |
| Hausa | 6 |
| Other African Language specify: | 7 |
| Other European or non-African language specify: | 8 |

**11. Please indicate the highest level of your parents’/guardians’ education**

|  | Father/ Guardian | Mother/ Guardian |
| --- | --- | --- |
| None/ No education | 1 | 1 |
| Primary education (some or complete) | 2 | 2 |
| Some secondary education but not completed | 3 | 3 |
| Completed secondary school (SSS/A’level) | 4 | 4 |
| A college diploma | 5 | 5 |
| A first degree | 6 | 6 |
| A postgraduate degree | 7 | 7 |

**12. At home, which parents or guardians do you live with?**

| Both my mother and father in the same household | 1 |
| --- | --- |
| Only my mother | 2 |
| My mother and stepfather | 3 |
| Only my father | 4 |
| My father and stepmother | 5 |
| Some of the time in my mother’s home and some in my father’s home | 6 |
| Other relatives (aunt, uncle, grandparent) | 7 |
| Guardian/foster parent who is not a relative | 8 |
| No parents or guardians (I live alone) | 9 |

**13. How would you describe your family’s socioeconomic status compared to other families in the area where you live?**

| We are a lot poorer than most | 1 |
| --- | --- |
| We are a little poorer than most | 2 |
| We have about the same amount of money as most | 3 |
| We are a little richer than most | 4 |
| We are a lot richer than most | 5 |

**14. What is your family’s religious affiliation? ***Single response***

|  | Self | Mother/Guardian | Fathe/Guardianr |
| --- | --- | --- | --- |
| Christian (Protestant) | 1 | 1 | 1 |
| Christian (Catholic) | 2 | 2 | 2 |
| Muslim | 3 | 3 | 3 |
| Judaism | 4 | 4 | 4 |
| Hinduism | 5 | 5 | 5 |
| Traditional African | 6 | 6 | 6 |
| No religious affiliation | 7 | 7 | 7 |
| Other: Specify (specify)......................................... | 8 | 8 | 8 |

**15. If affiliation is Protestant, which Denomination?**

|  | Self | Mother | Father | Guardian |
| --- | --- | --- | --- | --- |
| Anglican | 01 | 01 | 01 | 01 |
| Lutheran | 02 | 02 | 02 | 02 |
| Methodist | 03 | 03 | 03 | 03 |
| Presbyterian | 04 | 04 | 04 | 04 |
| Baptist | 05 | 05 | 05 | 05 |
| Quaker/Friends | 06 | 06 | 06 | 06 |
| Mennonite | 07 | 07 | 07 | 07 |
| Dutch Reformed | 08 | 08 | 08 | 08 |
| Calvinist | 09 | 09 | 09 | 09 |
| Evangelical | 10 | 10 | 10 | 10 |
| Pentecostal | 11 | 11 | 11 | 11 |
| Independent | 12 | 12 | 12 | 12 |
| Church of Christ | 13 | 13 | 13 | 13 |
| Zionist Christian Church | 14 | 14 | 14 | 14 |
| Jehovah’s Witness | 15 | 15 | 15 | 15 |
| Seventh Day Adventist | 16 | 16 | 16 | 16 |
| Mormon | 17 | 17 | 17 | 17 |

**16. How often do you attend church/mosque/synagogue/temple?**

| Never | 0 |
| --- | --- |
| Only on special occasions | 1 |
| Occasionally (several times a year) | 2 |
| Frequent attendance | 3 |
| Fairly Regular (Almost weekly) | 4 |
| Regular (Weekly) | 6 |

**17. How often does your family members (Father/Mother/siblings attend church / mosque / synagogue / temple etc.?**

| Never | 0 |
| --- | --- |
| Only on special occasions | 1 |
| Occasionally (several times a year) | 2 |
| Frequent attendance | 3 |
| Fairly Regular (Almost weekly) | 4 |
| Regular (Weekly) | 6 |

**18. How religious do you consider yourself to be?**

| Not religious at all | 1 |
| --- | --- |
| Somewhat religious | 2 |
| Moderately religious | 3 |
| Very religious | 4 |
| Extremely religious | 5 |

**19. How important is religion in your life?**

| Not at all important | 1 |
| --- | --- |
| Not very important | 2 |
| Somewhat important | 3 |
| Very important | 4 |
| Extremely important | 5 |
| Refused to answer | 8 |
| Don’t know (DNR) | 9 |

We will now ask you questions relating to your behaviour based on your response to question 21 above

**20. Please indicate your level of agreement to the following statements by circling the appropriate number that corresponds with the answer key.**

| Strongly Disagree | 1 |
| --- | --- |
| Disagree | 2 |
| Mostly disagree | 3 |
| Mostly Agree | 4 |
| Agree | 5 |
| Strongly Agree | 6 |

| a. I find meaning in my life experiences. | 1 | 2 | 3 | 4 | 5 | 6 |
| --- | --- | --- | --- | --- | --- | --- |
| b. I have a sense of purpose. | 1 | 2 | 3 | 4 | 5 | 6 |
| c. I am happy about the person I have become. | 1 | 2 | 3 | 4 | 5 | 6 |
| d. I see the sacredness in everyday life. | 1 | 2 | 3 | 4 | 5 | 6 |
| e. I meditate to gain access to my inner spirit | 1 | 2 | 3 | 4 | 5 | 6 |
| f. I live in harmony with nature. | 1 | 2 | 3 | 4 | 5 | 6 |
| g. I believe there is a connection between all things that I cannot see but can sense. | 1 | 2 | 3 | 4 | 5 | 6 |
| h. My life is a process of becoming. | 1 | 2 | 3 | 4 | 5 | 6 |
| i. I believe in a Higher Power/Universal Intelligence. | 1 | 2 | 3 | 4 | 5 | 6 |
| j. I believe that all living creatures deserve respect. | 1 | 2 | 3 | 4 | 5 | 6 |
| k. The earth is sacred. | 1 | 2 | 3 | 4 | 5 | 6 |
| l. I value maintaining and nurturing my relationships with others. | 1 | 2 | 3 | 4 | 5 | 6 |
| m.I use silence to get in touch with myself. | 1 | 2 | 3 | 4 | 5 | 6 |
| n. I believe that nature should be respected. | 1 | 2 | 3 | 4 | 5 | 6 |
| o. I have a relationship with a Higher Power/Universal Intelligence. | 1 | 2 | 3 | 4 | 5 | 6 |
| p. My spirituality gives me inner strength. | 1 | 2 | 3 | 4 | 5 | 6 |
| q. I am able to receive love from others. | 1 | 2 | 3 | 4 | 5 | 6 |
| r. My faith in a Higher Power/Universal Intelligence helps me cope during challenges in my life. | 1 | 2 | 3 | 4 | 5 | 6 |
| s. I strive to correct the excesses in my own lifestyle patterns/practices. | 1 | 2 | 3 | 4 | 5 | 6 |
| t. I respect the diversity of people. | 1 | 2 | 3 | 4 | 5 | 6 |
| u. Prayer is an integral part of my spiritual nature. | 1 | 2 | 3 | 4 | 5 | 6 |
| v. At times, I feel at one with the universe. | 1 | 2 | 3 | 4 | 5 | 6 |
| w. I often take time to assess my life choices as a way of living my spirituality. | 1 | 2 | 3 | 4 | 5 | 6 |

**21.** Given your religious or non-religious beliefs, h**ow often do you discuss POLITICS with each of the following people / groups? ***If category doesn’t apply e.g. if mother has passed away or is estranged, record NA (Not applicable)* ***1 response per row***

|  | **Never** | **Rarely** | **Sometimes** | **Often** | **Always** |
| --- | --- | --- | --- | --- | --- |
| Your father | 1 | 2 | 3 | 4 | 5 |
| Your mother | 1 | 2 | 3 | 4 | 5 |
| Family members other than parents | 1 | 2 | 3 | 4 | 5 |
| Friends/ classmates with different views | 1 | 2 | 3 | 4 | 5 |
| Friends and classmates with similar views | 1 | 2 | 3 | 4 | 5 |
| Members of political organisations on campus | 1 | 2 | 3 | 4 | 5 |
| Members of political organisations off campus | 1 | 2 | 3 | 4 | 5 |

**22. How often do you participate in the following activities? ***One response per row***

|  | **Never** | **Seldom rarely** | **Sometimes** | **Often** | **Always** |
| --- | --- | --- | --- | --- | --- |
| Attempting to influence the political views of others | 1 | 2 | 3 | 4 | 5 |
| Writing letters to the newspapers about political matters | 1 | 2 | 3 | 4 | 5 |
| Presenting your views to politicians (e.g. by signing petitions) | 1 | 2 | 3 | 4 | 5 |
| Participate in the activities of a political party | 1 | 2 | 3 | 4 | 5 |
| Participate in political protest marches/political sit-ins/demonstrations | 1 | 2 | 3 | 4 | 5 |
| Attending political rallies | 1 | 2 | 3 | 4 | 5 |
| Participate in the activities in the youth movement of a political party | 1 | 2 | 3 | 4 | 5 |
| Attending any mass meetings/rallies dealing with student politics on campus? | 1 | 2 | 3 | 4 | 5 |

**23. How important is it for students to participate in the following activities? ***One response per row***

|  | **Not at all important** | **Slightly Important** | **Neutral** | **Moderately Important** | **Very Important** |
| --- | --- | --- | --- | --- | --- |
| Elections for national government | 1 | 2 | 3 | 4 | 5 |
| Elections for local government | 1 | 2 | 3 | 4 | 5 |
| Student political associations | 1 | 2 | 3 | 4 | 5 |
| Community youth clubs | 1 | 2 | 3 | 4 | 5 |
| Environmental programmes | 1 | 2 | 3 | 4 | 5 |
| Community sanitation programmes | 1 | 2 | 3 | 4 | 5 |
| Neighbourhood safety activities | 1 | 2 | 3 | 4 | 5 |
| Sports and games | 1 | 2 | 3 | 4 | 5 |
| Other activities (specify)....................................... | 1 | 2 | 3 | 4 | 5 |

**24. Are you/did you participate in the following activities recently?**

|  | Yes | No |
| --- | --- | --- |
| a) Community youth club activities | 1 | 2 |
| b) Community environmental activities | 1 | 2 |
| c) Community sanitation programmes | 1 | 2 |
| d) Neighbourhood safety activities | 1 | 2 |
| e) Sports and games | 1 | 2 |
| f) Other activities (specify) ............ | | |

**25 To what extent do you feel comfortable about doing the following with somebody of a different ethnicity than your own? ***One response per row***

|  | **To no extent** | **To a small extent** | **To a medium extent** | **To a large extent** | **To a very large extent** |
| --- | --- | --- | --- | --- | --- |
| Attending lectures | 1 | 2 | 3 | 4 | 5 |
| Participating in a study group | 1 | 2 | 3 | 4 | 5 |
| Sharing accommodation but not the same room | 1 | 2 | 3 | 4 | 5 |
| Sharing a room | 1 | 2 | 3 | 4 | 5 |
| Being friends | 1 | 2 | 3 | 4 | 5 |
| Dating | 1 | 2 | 3 | 4 | 5 |
| Socialising with people from another ethnic group | 1 | 2 | 3 | 4 | 5 |
| Having friends who are members of a different ethnic group | 1 | 2 | 3 | 4 | 5 |

**26. How do you feel about the following statements? ***One response per row***

|  | **Strongly Disagree** | **Disagree** | **Neutral** | **Agree** | **Strongly Agree** |
| --- | --- | --- | --- | --- | --- |
| It is okay for people from different ethnic groups to attend social functions (parties, weddings, funerals etc.) together. | 1 | 2 | 3 | 4 | 5 |
| It is okay to associate mostly with your own ethnic group at a mixed social function. | 1 | 2 | 3 | 4 | 5 |
| It is okay for people from different ethnic groups to date each other. | 1 | 2 | 3 | 4 | 5 |
| People should be free to marry whoever they want to marry regardless of their ethnicity. | 1 | 2 | 3 | 4 | 5 |

Answer the following questions while taking into consideration your religious beliefs and convictions.

No

Yes

**27. Are you sexually experienced?**

**28. At what age did you become sexually active?**

No

Yes

**29. If sexually experienced, are you currently sexually active?**

**30. If currently sexually active, are you married or are you living with a man or woman as if married?**

No

Yes

**31. If not married, but sexually active, do you have a child you have given birth to or fathered?**

No

Yes

0

**32. How many children have you given birth to or fathered?**

1

2

3

4

5

6

Don’t Know

**33. If sexually experienced, how many times did you have sex in the last month?**

More than 4 times

At least 4 times

Less than 4 times

Once

Never had sex in the last moth

Don’t remember

**34. Have you ever used any of the following substances?**

| Marijuana | 1. Yes | 2. No |
| --- | --- | --- |
| Mandrax | 1. Yes | 2. No |
| LSD | 1. Yes | 2. No |
| Cocaine | 1. Yes | 2. No |
| Crack | 1. Yes | 2. No |
| Heroine | 1. Yes | 2. No |
| CAT | 1. Yes | 2. No |
| Ecstasy | 1. Yes | 2. No |
| Inhalants | 1. Yes | 2. No |
| Smoked cigarettes | 1. Yes | 2. No |
| Drunk any alcoholic beverages | 1. Yes | 2. No |

**35. Do you engage in any of the following behaviours currently?**

|  | Never | Sometimes | Always |
| --- | --- | --- | --- |
| Smoking cigarettes | 1 | 2 | 3 |
| Using illicit entertainment drugs such as marijuana | 1 | 2 | 3 |
| Drinking alcohol | 1 | 2 | 3 |
| Engage in violence against other people | 1 | 2 | 3 |

**36. How do you feel about the following statements?**

|  | Strongly agree | Disagree | Neutral | Agree | Strongly disagree |
| --- | --- | --- | --- | --- | --- |
| It is okay for young people to engage in premarital sex. | 1 | 2 | 3 | 4 | 5 |
| It is okay for young people to engage in premarital childbearing. | 1 | 2 | 3 | 4 | 5 |
| It is okay for young people to use drugs. | 1 | 2 | 3 | 4 | 5 |
| It is okay for young people to consume alcohol. | 1 | 2 | 3 | 4 | 5 |
| It is okay for young people to engage in violence for any reason | 1 | 2 | 3 | 4 | 5 |

**THANK YOU FOR YOUR COOPERATION**
